# Patient information, education and self-management in bronchiectasis: facilitating improvements to optimise health outcomes

**DOI:** 10.1186/s12890-018-0633-5

**Published:** 2018-05-22

**Authors:** Katy L. M. Hester, Julia Newton, Tim Rapley, Anthony De Soyza

**Affiliations:** 1 Institute of Cellular Medicine, Newcastle University, Newcastle upon Tyne, NE2 4HH UK; 2 Adult Bronchiectasis Service, Freeman Hospital, Newcastle upon Tyne, NE7 7DN UK; 3 Faculty of Medical Sciences, Newcastle University, Newcastle upon Tyne, NE2 4HH UK; 4 Department of Social Work, Education and Community Wellbeing, Northumbria University, Newcastle upon Tyne, NE7 7XA UK

**Keywords:** Bronchiectasis, Self-management, Information, Education, Qualitative, Adherence

## Abstract

**Background:**

Bronchiectasis is an incurable lung disease characterised by irreversible airway dilatation. It causes symptoms including chronic productive cough, dyspnoea, and recurrent respiratory infections often requiring hospital admission. Fatigue and reductions in quality of life are also reported in bronchiectasis. Patients often require multi-modal treatments that can be burdensome, leading to issues with adherence. In this article we review the provision of, and requirement for, education and information in bronchiectasis.

**Discussion:**

To date, little research has been undertaken to improve self-management in bronchiectasis in comparison to other chronic conditions, such as COPD, for which there has been a wealth of recent developments. Qualitative work has begun to establish that information deficit is one of the potential barriers to self-management, and that patients feel having credible information is fundamental when learning to live with and manage bronchiectasis. Emerging research offers some insights into ways of improving treatment adherence and approaches to self-management education; highlighting ways of addressing the specific unmet information needs of patients and their families who are living with bronchiectasis.

**Conclusions:**

We propose non-pharmacological recommendations to optimise patient self-management and symptom recognition; with the aim of facilitating measurable improvements in health outcomes for patients with bronchiectasis.

## Background

Bronchiectasis is a chronic lung condition that leads to a significant symptom and treatment burden for those affected, and significant costs to healthcare services such as the National Health Service. Worldwide prevalence is increasing [1–3], yet the evidence base for the management of bronchiectasis remains poor [4, 5]. Historically, there has been relatively little research conducted in this field and it is only recently that more attention has been paid to this previously somewhat neglected disease. Improvements in disease management interventions in bronchiectasis are required.

Interventions in bronchiectasis are likely to include better medical therapies, yet it is also apparent that bronchiectasis is a ‘model’ chronic disease in terms of its potential for improvements in self-management. If patients and their carers know how to recognise symptoms of deterioration or exacerbation, and know how and when to take action, this could facilitate improvements in self-management. This in turn has the potential to promote disease stability, reductions in unscheduled presentations to acute health care services and improvements in longer-term health.

Whilst clearly desirable, expecting patients to understand their condition, the treatments used and the implications of not using them appropriately, is potentially challenging. Prior work has demonstrated that patients with bronchiectasis feel more confident with their treatments when they have received information about them in a specialist clinic [6]. Not every patient with bronchiectasis, however, has access to such information or specialist expertise. Despite recommendations for education and personalised management plans for patients with bronchiectasis [4, 7], there remains a lack of information material openly available to patients when compared to other chronic medical and respiratory conditions. For example, patients with chronic obstructive pulmonary disease (COPD) have a number of available resources [8–12]. In order to facilitate self-care, patients need to have accurate and accessible information about their condition, enabling them to recognise and respond to triggers appropriately and understand how their use of self-management could potentially alter their prognosis. Suitable education could lead to a level of self-management that results in clinically and biologically important endpoints in bronchiectasis. In this article we review the need for education and information in bronchiectasis and its current provision. We discuss options for future improvements in resource development to facilitate much needed improvements in health outcomes.

## The burden of bronchiectasis: symptoms, prevalence and treatments

Bronchiectasis leads to symptoms of breathlessness, cough and a chronic infective syndrome and consequently, a poorer quality of life [13, 14] and clinically significant fatigue [15, 16]. Patients often have recurrent infective exacerbations, some of which result in costly hospital admissions. Patients with bronchiectasis are at an increased risk of anxiety and depression [17–19]. In cystic fibrosis, depression and anxiety rates are higher than in the general population, and therefore annual screening is recommended [20, 21]. Given the potential for such psychological distress to impact upon adherence and disease management, annual screening in bronchiectasis could also be of benefit. New data has additionally shown a greater risk of coronary heart disease and stroke in patients with bronchiectasis [22]. Multiple comorbidities are common in bronchiectasis [23]. The burden of disease for patients and carers is clearly significant.

The burden of bronchiectasis for healthcare services is also significant. Recently, UK data reported a prevalence of between 43.4/100,000 in those aged 18–30 and 1239.7/100,000 in those aged 70–79 [1]. There is further evidence that prevalence is increasing worldwide [2, 3]. Additionally, HRCT imaging studies report that up to 50% of patients with COPD have co-existent bronchiectasis [24, 25] and it has been proposed that COPD and bronchiectasis can co-exist as ‘bronchiectasis COPD overlap syndrome’ (BCOS) [26]. With approximately 1,000,000 patients with a diagnosis of COPD in the UK alone, [27] there is potential for the rise in prevalence of both bronchiectasis and BCOS to continue.

Bronchiectasis mortality rates have been reported in the UK as twice that of the general population [1], approximately 50% higher than that of uncomplicated COPD (calculated at 3% per annum) and are increasing [28, 29]. The presence of BCOS also leads to higher mortality rates [30–32]. Prognosis varies in bronchiectasis, with a study of 91 patients finding that the primary cause of death was usually respiratory, with survival rates of 91% at 4 years and 68.3% at 12.3 years [33]. The same study found factors such as chronic infection with *Pseudomonas aeruginosa* increase mortality. Two severity scores have been recently developed for use in bronchiectasis: the bronchiectasis severity index (BSI) [34] and the FACED score [35]. Although both predict mortality, the BSI is also predictive of severe exacerbations, hospital admissions and quality of life [36, 37]. Infective exacerbations lead to significant morbidity in bronchiectasis. The British Thoracic Society (BTS) national bronchiectasis audit reported that 38% of patients had three or more exacerbations per year [38]. Within cohorts of patients across Europe, reported average exacerbation rates have been from 1 to 4 per year [36, 39]. This is also consistent with American data on the increasing burden of bronchiectasis [40].

Earlier UK data also emphasised the burden of bronchiectasis, uncertainties in aetiology and lack of evidence for the treatments that are often used [41]. Although there are guidelines for investigation, diagnosis and management of bronchiectasis produced by the BTS, there is no cure for bronchiectasis and many therapies are empiric and not evidence based [4]. Bronchiectasis differs from some chronic diseases in both its periods of exacerbation and the role patients may play in managing these. In bronchiectasis, correct, timely recognition of exacerbation symptoms and prompt, appropriate management of infections could lead to increased disease stability. Failure to commence antibiotics promptly could result in a more severe exacerbation of bronchiectasis, potentially requiring hospital admission. This would lead to significant additional healthcare costs, and a much greater physical, psychological and social impact on patients and their families. Conversely, inappropriate and excessive antibiotic use can lead to antibiotic resistance. This could also have problematic repercussions in terms of response to future treatments and consequently longer-term health outcomes. Facilitating patients’ understanding and ability to self-manage is therefore extremely important.

With some exceptions, treatments are broadly similar regardless of the aetiology of bronchiectasis, but specific treatment plans are tailored to the individual. These can range from no regular treatments at all, to daily use of nebulised therapies in conjunction with physiotherapy, inhalers and oral medications that collectively can be significantly complex and time-consuming for patients and their families. There is evidence that adhering to inhaled antibiotics decreases exacerbation rate and that poor adherence is associated with poorer outcomes [42, 43]. Understanding the importance of a variety of treatments can be problematic for patients and their families and suitable information and education is required to encourage adherence to mutually agreed treatment plans. Importantly, it is known that those with more frequent exacerbations suffer not only the physical effects, but also a reduction in quality of life [14]. Measuring how the psychological impacts and co-morbidities of bronchiectasis affect treatment adherence and self-management behaviours is a key challenge.

## Current provision of information for patients with bronchiectasis

The education of patients with bronchiectasis is recommended; including explanations of the disease, recognition and importance of exacerbations, treatment approaches and a personal management plan [4, 7]. Although patients with bronchiectasis gain information through discussion with their clinician, there is relatively little additional information available to them in comparison to the number of resources available for other chronic conditions such as COPD or Cystic Fibrosis. When searching online for information about bronchiectasis, there are resources available. Some are produced by governmental bodies (e.g. the UK National Health Service (NHS) and the US National Institutes of Health), others by health information providers, and by hospitals and charitable organisations [44–48]. However, in addition to being limited in number when compared to other conditions, many are either very brief, with limited information provided, or are lengthy and potentially overwhelming. There are some resources run by patients which primarily serve as a patient’s view or online forum rather than an information resource per se [49, 50]. To date there are no high quality trials nor systematic reviews of such information provision in bronchiectasis, as conducted in COPD [8–12, 51].

A tabulated summary (Table 1) shows examples of some of the bronchiectasis information resources that are available online. Determining credibility (affiliation with a well-known lung charity or a national healthcare provider, for example) is a necessary step in the selection process for patients seeking health information [52]. Despite the available information online, patients and carers with bronchiectasis have reported a lack of trustworthy and user-friendly information and felt they would benefit from credible, multi-format (text, images and video content) resources that they could continue to access outside of a clinic setting [53]. Further identification of the unmet information needs of affected individuals and preferred information formats would enable appropriate resource development. A key priority is to create an accessible, trustworthy resource containing information that users want, rather than information that providers have decided patients should have.

**Table 1:** A selection of available online bronchiectasis patient information resources (English language)

Resource	Provider	Description	User Co-production	Recognised healthcare service provider, academic organisation or charity?	Information in video format?
www.bronchiectasis.me	Produced by a multidisciplinary bronchiectasis team and patients and carers based at Newcastle University and Newcastle upon Tyne Hospitals NHS Foundation Trust	Content and format based upon findings of qualitative research with patients and carers. Comprehensive information in text (13,000 words. Sub-sectioned so small, relevant portions of information visible at a time) plus images and multiple videos. Option to download PDF. Updated and available open access June 2017	Yes. Co-produced with patients and carers and based upon prior research.	Yes	Yes
http://www.bronchiectasis.scot.nhs.uk/	NHS Lothian and input from patients with bronchiectasis	Online information with input from patients (Approximately 3000 words plus written patient stories). Option to download PDF brochure. Last updated Jan 2015.	Yes. User input during development.	Yes	No
https://www.blf.org.uk/support-for-you/bronchiectasis	British Lung Foundation (BLF)	Online and booklet version available. Previously quite brief information about bronchiectasis (900 words). Revised June 2017 to include more comprehensive information in keeping with the resources used in the BRIEF study [108].	User reviewers	Yes	No
http://www.nhs.uk/Conditions/Bronchiectasis/Pages/Diagnosis.aspx	U.K. National Health Service	Online information (3600 words). Lots of text on the page to scroll through plus extra items alongside. Last updated 2015	Not specified	Yes	No
http://patient.info/health/bronchiectasis-leaflet	Patient (patient and professional information provider)	Online text. Multiple adverts alongside. Includes discussion forum. Last Updated 2014	Not specified	No	No
http://www.bronchiectasishelp.org.uk/	Written by a patient with bronchiectasis	Patient’s perspective with input from professionals. Not text-heavy. No Adverts.	Yes	No	No
https://www.nhlbi.nih.gov/health/health-topics/topics/brn	National Heart Lung and Blood Institute (USA)	Well organised sections. High volume of information and text (4500 words). No Adverts. Last updated 2014	Not specified	Yes	No
http://www.bronchiectasis.info/	Run by patients with bronchiectasis	Discussion forum and online community rather than an information resource.	Yes	No	No
https://en.wikipedia.org/wiki/Bronchiectasis	Wikipedia	Text and images. Last edited April 2017	Not specified	No	No
https://medlineplus.gov/ency/article/000144.htm	US National Library of Medicine	Concise information (570 words). Last updated April 2017.	Not specified	Yes	No
	British Medical Journal	Concise information (1200 words) no adverts. All text format, no pictorial content. Last updated October 2015.	Not specified	Yes	No
	BLF online presentation and question and answers with consultant and physiotherapist.	YouTube video of presentation slides and audio. Patient questions and answers. Published 2012.	Not specified	Yes	Yes
http://www.webmd.boots.com/a-to-z-guides/bronchiectasis	Boots, Pharmacy/ WebMD. Last reviewed July 2015.	Online text information (1300 words). Lots of adverts on the page. Last updated July 2015.	Not specified	No (*)	No

(*) Provider notes: **“**subject to rigorous review and approval by doctors in the UK” is noted in the home page of the overall site but not noted on bronchiectasis specific pages

## Provision of information for chronic conditions: General principles and its relationship to self-management

The importance of information provision for patients with long-term conditions and their carers is well recognised. There is longstanding evidence that patients want to access information [54] and that information can reduce anxiety [55] and improve patient outcomes [56, 57]. Self-management is increasingly recognised as an important part of chronic disease management and is recommended by the World Health Organisation (WHO) [58]. Information provision is key to facilitating self-care [59] and inadequacies in information provision are a potential reason for people managing their health poorly [60]. Information provision therefore plays an important role in supporting active participation in care and remains a priority area of health research for all chronic conditions.

Arguably, however, information alone does not always translate into behavioural change [61]. Providing patients with a simple factsheet about their condition is unlikely to result in any major tangible benefits. In a review of the role of education in asthma, it was recognised that information about asthma should not simply be factual but allow patients to acquire skills [62]. It is preferable to teach patients about their asthma treatments and inflammation rather than the structure and function of the lungs, for example [63]. Theoretical constructs and behavioural change techniques beyond information delivery are important to consider in development of any intervention that aims to produce changes in behaviour [64, 65]. Framing information, by establishing what is relevant to the patient group, and how it could be delivered in order to achieve the desired effects is therefore essential. For example, video demonstrations or instructions on how to perform certain tasks in bronchiectasis management such as chest clearance has been identified by patients as a priority [53]. This provision of instruction and demonstration of behaviours is in keeping with social-cognitive theory [66]. Information on behaviour-health links and consequences of actions or inactions, would be in keeping with an information-motivation-behavioural skills model [67]. Information is an intervention, the production of which is a highly skilled process and ideally resources should be user tested, co-designed and co-produced where possible [68]. Such an approach should be taken to resource development for bronchiectasis [69].

Self-management and patient information provision are seemingly inextricably linked. In primary care, self-management has been referred to as ‘patients with chronic disease making day to day decisions about their illnesses’ [70] and ‘the everyday tasks and activities that a person living with a chronic condition needs to carry out’ [71]. The aim in supporting self-management is to allow patients to gain not only the knowledge but also the confidence and relevant skills to manage their condition; promoting patient ‘activation’ [72, 73]. An important concept embedded within this is self-efficacy: the confidence that one can carry out a behaviour necessary to achieve a desired goal [66]. Using self-efficacy as a measurable outcome, however, is not without flaws, as was shown when trialling the expert patient programme (EPP) [74–76]. The EPP was initially developed for patients with arthritis and designed to enhance disease specific information rather than replace it, as the programme is generic in nature. Although these studies found improvements in symptom control, pain and hospitalisations as well as gains in self-efficacy, there are some criticisms of the EPP. These include self-efficacy gains not leading to improvements in self-management and not necessarily reducing hospital presentations. Additionally, it has been suggested that participants in EPP studies were not representative of the general population and were possibly better at self-managing than most [77–79]. A UK study using a lay-led EPP (*n* = 629) showed an increase in participant self-efficacy and energy, yet no reduction in health care utilisation [80].

A number of self-management educational resources have been produced for chronic lung conditions other than bronchiectasis. For example, SPACE for COPD, a six week intervention [51]. At 6 months, there were gains in disease knowledge, anxiety and performance levels, yet the primary outcome measure of dyspnoea had not improved [9]. Living Well with COPD [10] is a website with information and videos requiring a password obtained by patients from their physician in order to access the full material. A two year randomised controlled trial conducted in primary care did not show long term benefits over usual care when using measures of self-efficacy and quality of life. The group with access to the living well programme, however, did seem more able to manage their exacerbations [81]. In asthma, a study using an educational programme based on repeated short interventions (face to face sessions at 3 month intervals over 1 year, a personalised action plan and inhaler technique training) saw improvements in asthma control in the intervention group, yet a degree of improvement within the control group was noted too [82]. Cost effectiveness was not examined and although the intervention was brief, it would involve staff time at considerable cost, and there was no provision for patient information needs at other time points.

Systematic reviews of such self-management education in chronic lung disease have also been conducted. A review of eight studies in COPD revealed inconclusive evidence of any benefits [12]. A more recent review and meta-synthesis, however, concluded that self-management education could reduce hospital admissions in COPD, and improve disease knowledge and quality of life. It did not lead to reductions in mortality or smoking rates, nor improve dyspnoea or lung function [11]. A recent systematic review and meta-analysis across different disease areas concluded that self-management interventions can be implemented without a detrimental effect on health outcomes and that they reduce service utilisation [57]. Although the effect sizes were small overall, it is of note that respiratory conditions were amongst the two groups that had the strongest evidence of benefit. In asthma, a Cochrane review reported that self-management education could improve health outcomes only when delivered in conjunction with medical reviews and a written action plan [83]. In cystic fibrosis, there were too few data to draw firm conclusions about recommendations for self-management [84]. In addition, a separate review of psychological interventions in cystic fibrosis highlighted that behavioural interventions had some effect in improving nutrition, and decision making tools regarding transplantation improved knowledge and expectations of transplant, yet there remained insufficient evidence overall [85]. A protocol for a systematic review of self-management in non-cystic fibrosis bronchiectasis has recently been published [86]. Conclusions are likely to be limited, however, reflecting the limited available literature on self-management in bronchiectasis.

## Facilitating self-management in bronchiectasis: Use of information and considerations for resource development

An evidence-based intervention for use in bronchiectasis is still needed. In a study using focus groups, patients with bronchiectasis perceived lack of information and confidence as barriers to self-management and felt that disease specific information would be useful [87]. The use of an EPP as part of a self-management programme for patients with bronchiectasis has also been investigated [88]. The programme consisted of two group sessions of disease-specific information followed by a standard, generic EPP for six weeks. Improvement was found in six of ten domains of a self-efficacy scale, including managing symptoms and depression. The intervention group, however, also reported more symptoms and reduced quality of life post intervention. The educational sessions about bronchiectasis were not patient-driven in terms of content or format of delivery, and participants commented that the sessions should be condensed and be attended by physicians. Costs, staffing, time and patient commitment involved with such a course are considerable, making it potentially unfeasible to deliver at scale within a clinical setting. A successful intervention for bronchiectasis would need to meet patients’ needs, be easily accessible and be feasibly deliverable on a long-term basis.

Another recent study has taken a different approach to aiding self-management in patients with bronchiectasis, using a novel tool, the Bronchiectasis Empowerment Tool [89]. The tool consisted of a one page action plan, within a pack containing information and optional notepads. The reported aim of the study was to work alongside existing care in order to improve self-management. At the time of writing the study is closed to recruitment but no published results are available.

In bronchiectasis, self-management includes making decisions surrounding adherence to treatments. Factors predicting adherence to treatments could include beliefs about treatments and burden of treatment [90]. Based on these findings, a theoretical approach is being used to work towards development of a behaviour change intervention to promote treatment adherence [91]. The need for information was again reported by patients during interviews. Participants thought that having knowledge about bronchiectasis and treatments improved adherence.

There is evidence, in COPD, that gaps in knowledge of health care professionals can impact upon patients’ knowledge and understanding of their condition [92]. This finding may apply to other conditions. Patients who have bronchiectasis may not attend a specialist service, and general physicians may have less disease-specific knowledge than a specialist delivering a bronchiectasis service. In addition, they may not have sufficient exposure to have developed specific expertise in exchanging disease-specific information in a patient-focussed manner. Given this relative lack of exposure to patients who have bronchiectasis, further development of such skills in an area outside a healthcare providers’ area of main expertise is likely to be problematic. In exploratory interviews with patients who had bronchiectasis, [52] participants referred to the fact that they had very little information until they started to attend a specialist bronchiectasis clinic. A trustworthy and patient-driven resource would enable both dissemination of good practice and equity of information access amongst the patient group.

Information seeking is another important aspect to consider in developing an understanding of patients’ information needs and how such needs are fulfilled. Trust, particularly in relation to online resources, is a recognised issue [93, 94]. Reasons identified for avoiding seeking information in patients with bronchiectasis include not trusting sources, and fear of what they may find [52]. The potential for information to worsen rather than reduce anxiety has been proposed [95], and the concept of information avoidance is recognised [96]. Reviews of health information seeking behaviour have concluded that a better understanding of this concept will enable the provision of better information, and that information should meet patients’ individual needs [95, 96]. Previous work exploring why patients with cancer may not want or seek information about their condition also identified that patients’ attitudes and coping strategies can limit their seeking of information [97]. The importance of identifying the diversity of needs of the patient group in order to tailor resources to suit them rather than assume a ‘one size fits all’ approach will be effective was additionally highlighted. Having an in depth understanding of the information and education needs of both patients and their families, and how these could be met, in addition to an appreciation of how, why and when patients seek information would appear to be fundamental to the development and execution of novel interventions in bronchiectasis. Understanding these needs across a broad sample of potential users with differing backgrounds, ages and disease severities, for example, is also important. Those with bronchiectasis aetiologies that are associated with additional management challenges or poorer outcomes, for example, non-tuberculous mycobacterium infections, bronchiectasis-COPD overlap syndrome (BCOS) [26] and bronchiectasis rheumatoid arthritis syndrome (BROS) [98] may benefit from supplementary educational resources specific to their needs.

Patients use information to aid their decision making about various aspects of their management. Carers are often involved in shared decision-making in a variety of different ways and patients are rarely entirely autonomous in these processes [99, 100]. The role of family or carers in the adaptation of patients and coping with chronic illness is very important [101, 102]. Carers are just as likely to engage with information resources as patients. Therefore, any newly developed resource should accommodate the needs of carers and families of patients with bronchiectasis in addition to patients. A meta-synthesis of qualitative studies highlighted the importance of social networks (family, friends, communities) in the self-management of chronic illnesses [103]. A longitudinal study of patients with heart disease and diabetes also acknowledges this role of social networks in supporting self-management [104]. Participation in community organisations (including online communities and health education groups) has been associated with better physical and mental health within the self-management of diabetes [105, 106]. It is apparent that when considering educational and self-management support interventions they need to be tailored to users’ approaches.

Work has begun in bronchiectasis, as yet published only in abstract form, to identify the unmet needs of patients and carers living with bronchiectasis, and co-develop a multi-format information resource [53, 107]. A feasibility study carried out using a resource developed with users, based on their needs and experiences, included user evaluations of the resource and web analytics [108]. A particular feature of this resource is the use of video in information delivery, from professionals, patients and carers. This use of video was based on the findings of prior qualitative interviews and was found to be a particularly engaging aspect of the resource [69, 109]. The website (www.bronchiectasis.me) had over 27,000 worldwide views during the study period of 16 months. By delivering interventions that have been designed in conjunction with patients, to complement their learning approaches, information resources can be tailored to meet needs and optimise uptake.

## Conclusions

Patients and carers living with bronchiectasis have been relatively poorly provided for in terms of health information and self-management guidance, despite clear potential for such interventions to produce tangible benefits for patients and health care service providers. The prevalence of bronchiectasis is rising and makes this issue ever more pressing. This lack of provision should be addressed with a patient-centred approach, incorporating knowledge of information seeking and self-management in both other chronic diseases and bronchiectasis itself. In order to achieve development of patient-driven and user-friendly resources, the underlying needs and issues surrounding information and its uptake for patients with bronchiectasis must first be fully identified. The implementation of systematic annual screening for depression and anxiety could also identify patients with requirements for additional psychological support.

As with information delivered in a clinic setting, patients’ needs vary. By ensuring the involvement of diverse groups during co-development processes, this range of views can be captured. By developing resources with clearly labelled and sub-sectioned subject areas, users can interact with the information they need, when they need it, and avoid what they may not need or want to know. Using healthcare experts across the multidisciplinary team, patients and carers to co-produce high quality information and education resources is an important step towards facilitating self-management advancements, improvements in adherence and consequent physical and psychological health improvements in bronchiectasis.

## Abbreviations

BCOS: Bronchiectasis COPD overlap syndrome
BLF: British Lung Foundation
BROS: Bronchiectasis rheumatoid arthritis syndrome
BSI: Bronchiectasis severity index
BTS: British Thoracic Society
COPD: Chronic obstructive pulmonary disease
EPP: Expert patient programme
HRCT: High resolution computed tomography
NHS: National Health Service
UK: United Kingdom
WHO: World Health Organisation
